# Growth and Asymmetry of Soil Microfungal Colonies from “Evolution Canyon,” Lower Nahal Oren, Mount Carmel, Israel

**DOI:** 10.1371/journal.pone.0034689

**Published:** 2012-04-16

**Authors:** Shmuel Raz, John H. Graham, Ayelet Cohen, Benjamin L. de Bivort, Isabella Grishkan, Eviatar Nevo

**Affiliations:** 1 Department of Evolutionary and Environmental Biology, Institute of Evolution, University of Haifa, Haifa, Israel; 2 Department of Biology, Berry College, Mount Berry, Georgia, United States of America; 3 Rowland Institute at Harvard, Harvard University, Cambridge, Massachusetts, United States of America; National Cancer Institute, United States

## Abstract

**Background:**

Fluctuating asymmetry is a contentious indicator of stress in populations of animals and plants. Nevertheless, it is a measure of developmental noise, typically obtained by measuring asymmetry across an individual organism's left-right axis of symmetry. These individual, signed asymmetries are symmetrically distributed around a mean of zero. Fluctuating asymmetry, however, has rarely been studied in microorganisms, and never in fungi.

**Objective and Methods:**

We examined colony growth and random phenotypic variation of five soil microfungal species isolated from the opposing slopes of “Evolution Canyon,” Mount Carmel, Israel. This canyon provides an opportunity to study diverse taxa inhabiting a single microsite, under different kinds and intensities of abiotic and biotic stress. The south-facing “African” slope of “Evolution Canyon” is xeric, warm, and tropical. It is only 200 m, on average, from the north-facing “European” slope, which is mesic, cool, and temperate. Five fungal species inhabiting both the south-facing “African” slope, and the north-facing “European” slope of the canyon were grown under controlled laboratory conditions, where we measured the fluctuating radial asymmetry and sizes of their colonies.

**Results:**

Different species displayed different amounts of radial asymmetry (and colony size). Moreover, there were highly significant slope by species interactions for size, and marginally significant ones for fluctuating asymmetry. There were no universal differences (i.e., across all species) in radial asymmetry and colony size between strains from “African” and “European” slopes, but colonies of *Clonostachys rosea* from the “African” slope were more asymmetric than those from the “European” slope.

**Conclusions and Significance:**

Our study suggests that fluctuating radial asymmetry has potential as an indicator of random phenotypic variation and stress in soil microfungi. Interaction of slope and species for both growth rate and asymmetry of microfungi in a common environment is evidence of genetic differences between the “African” and “European” slopes of “Evolution Canyon.”

## Introduction

Fluctuating asymmetry (FA) consists of random, typically small, unbiased deviations from perfect symmetry [1], [2], [3]. It is a widely used indicator of environmental and genetic stress [4], [5], [6], [7], [8], [9], [10] and is a measure of developmental instability, the failure of an individual to both correct fluctuations and buffer developmental noise [5], [11], [12]. For evolutionary biologists, fluctuating asymmetry reflects a population's state of adaptation and average fitness, where higher deviations from perfect symmetry correspond to higher stress and lower fitness [13], [14]. Consequently, it is a potentially useful indicator of disturbance, stress, and ecosystem change.

Most studies of fluctuating asymmetry focus on deviations from perfect bilateral symmetry in plants and animals [2], [5], [11]. A few studies have addressed helical, radial, and translatory symmetry [5], [15], [16], [17]. To our knowledge, however, there have been no explicit studies of fluctuating asymmetry in any microorganism, despite their potential application [15]. This presents an opportunity, because many species belonging to the kingdoms Fungi and Bacteria display morphological symmetry, and radial symmetry in particular.

**Figure 1:**
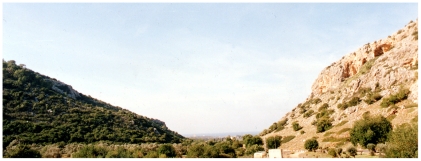
The opposing slopes of “Evolution Canyon” I, Lower Nahal Oren, Mount Carmel. The xeric “African” slope is on the right, and the mesic “European” slope is on the left (Raz et al., 2009).

We therefore performed the first study of fluctuating radial asymmetry in a microorganism. In this paper, we examine fluctuating radial asymmetry of five species of soil microfungi isolated from contrasting environments at “Evolution Canyon,” Lower Nahal Oren, Mount Carmel, Israel (Figure 1). Using standardized sampling and measurement (see “morphological measurement” in the Material and Methods section for additional information), applied to several species that share the same microsite, we extend the study of developmental instability to soil microfungi.

Like other organisms with indeterminate growth, fungi do not have a genetically determined limit of upper size and can potentially continue their growth indefinitely when environmental conditions are favorable (e.g., [18]). In soil, fungal colonies grow as an interconnected network of filamentous hyphae through the pore channels (i.e., interstices) of the soil [19]. Their growth is apical, by means of the hyphal tips, and the majority of resources are gained through these tips [20]. The dynamics of fungal colony growth are complex and determined by microclimate and the physico-chemical properties of the soil, as well as interactions with other soil-inhabiting organisms including other fungi [21]. Since it is impossible to measure the size and asymmetry of soil microfungal colonies in the field, we measured them in a controlled environment, where we can hold constant external factors such as humidity, medium composition, and temperature (e.g., [22], [23]). Specifically, cultures were grown on nutrient agar medium, where fungi typically form roughly circular colonies, whose symmetry can be measured quantitatively.

The adaptive architecture of fluctuating asymmetry is unknown. Moreover, the heritability of fluctuating asymmetry is usually, but not always [24], insignificant, and close to zero [25], [26]. Estimates of the heritability of fluctuating asymmetry require large sample sizes (reviewed in [5]). Finally, epistatic interactions may also contribute to genetic variation of fluctuating asymmetry [5], [25], [27].

Taking into account the complexity of interactions between soil microfungi and their heterogeneous environments, we inferred differential adaptation to the sharply contrasting habitats at “Evolution Canyon” by examining fungal developmental stability and growth rate in a common-garden experiment. If microfungi from the two slopes are not differentially adapted, then there should be no differences in growth and asymmetry between the slopes. Moreover, the interaction between slope and species should be insignificant as well. However, the converse does not necessarily hold—strains growing similarly in the lab may yet be differentially adapted in the wild.

“Evolution Canyon” (Nevo list at http://evolution.haifa.ac.il) is located at lower Nahal Oren (32°42′51.09″N; 34°58′26.81″E), a deeply incised valley (Figure 1) running from Mount Carmel, Israel, westwards to the Mediterranean Sea. The opposite slopes of “Evolution Canyon” are the south-facing, “African” slope and the north-facing, “European” slope. These two slopes are dramatically different, both biotically and abiotically, and provide a rare opportunity for studying developmental instability in a natural experiment. Interslope distance is 100 m at the valley bottom and 400 m at the top. “African” and “European” slopes are 120 m and 180 m long, respectively. The percentage of plant cover varies from 35% on the “African” slope to 150% on the “European” slope [28].

The opposite slopes of the canyon have identical geology and soil, but are covered by different vegetation: savannoid, open park forest on the “African” slope, and dense, south-European macquis forest on the “European” slope. Microclimate is the major abiotic interslope difference [29]. The “African” slope is more stressful for many ‘mesic’ organisms whereas the “European” slope is more stressful for many ‘xeric’ organisms (reviewed in [30], [31], [32], [33], [34] and [35], [36]). The microclimatic differences produce strong local differentiation at all biological levels: allozyme frequencies, DNA sequences, genes, genomes, populations, species, ecosystems, and biota [30], [31], [32], [33], [34]. Interslope differences at the molecular level (e.g., higher mutation frequency and recombination rate on the “African” slope in different taxa) are accompanied by interslope differences in species richness and abundance (reviewed in [30], [31], [32], [33], [34]).

Two fluctuating asymmetry studies were previously conducted in-situ on animal species from “Evolution Canyon.” Derzhavets et al. [37] found greater fluctuating asymmetry of *Drosophila melanogaster* wings on the “African” slope. Low humidity on this slope was a likely stressor for this species [38]. Raz et al. (unpublished report) studied the grain beetle *Oryzaephilus surinamensis*. This species was more abundant on the “European” slope, but showed no significant differences in fluctuating asymmetry between the slopes.

One expects that varying adaptations of fungi to different insolation, temperature, and humidity on the opposite slopes of “Evolution Canyon” will influence growth and developmental stability in a long-lasting, heritable way, manifested even when individual colonies are grown under common-garden conditions in the laboratory. The laboratory environment has a comparatively moderate temperature and humidity and probably resembles the “European” slope more than the “African” slope. Therefore, populations adapted to higher temperatures and lower humidity on the “African” slope should grow more slowly and be developmentally more unstable under this experiment, while populations adapted to lower temperatures and greater humidity on the “European” slope should grow more quickly and be developmentally more stable in the common garden. A previous study [9] of leaf asymmetry of twelve species of vascular plants growing at “Evolution Canyon” found that differences in fluctuating asymmetry between the two slopes were negatively correlated with differences in local abundance; species displayed higher fluctuating asymmetry on the slope where they were less abundant, i.e., under higher stress.

## Materials and Methods

### Sampling and isolation of microfungi

**Figure 2:**
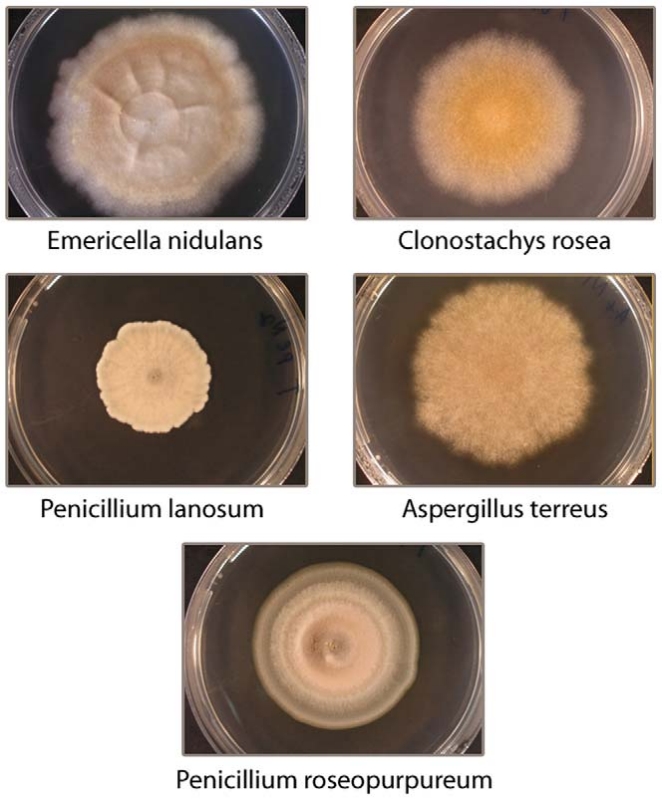
The soil microfungus species.

Ten soil samples were collected from the upper 1–5 cm of soil on each slope of “Evolution Canyon,” during January 2009. No specific permissions were required for collecting soil at this location (Lower Nahal Oren) The location is not privately-owned or protected in any way and the field studies did not involve endangered or protected species. Microfungi were isolated from the samples using the soil dilution plate method [39]. We were able to cultivate five species inhabiting both slopes: *Emericella (Aspergillus) nidulans*, *Aspergillus terreus*, *Penicillium lanosum*, *P. roseopurpureum*, and *Clonostachys rosea* (Figure 2). Species were identified based on morphological characteristics of fungal colonies. All five microfungal species are cosmopolitan, with worldwide distributions (e.g., [40]). *Clonostachys rosea* is a common fast-growing soil and rhizosphere fungus [40]. Other species represent both the thermo-tolerant soil microfungi (*Aspergillus* spp.), more frequently occurring in warm, xeric regions, and the mesophilic soil microfungi (*Penicillium* spp.), which are characteristic of cool-temperate mycobiotas.

The relative abundance of species in a sample was calculated by dividing the number of colonies of the species by the total number of colonies in the sample. The relative abundance of each species on a slope was estimated by averaging their abundances in the ten soil samples. For each species, we isolated from the different soil samples 4–10 parental colonies (i.e., strains), presumably genetically distinct. We sampled 2–3 clonal replicates by picking cells from the isolated parental colony. The fungal colonies were grown on 90-mm diameter agar plates with Czapek Agar at a temperature of 25°C. We seeded one colony in the center of each plate for 96 hours growth for all the study species.

### Morphological measurement

**Figure 3:**
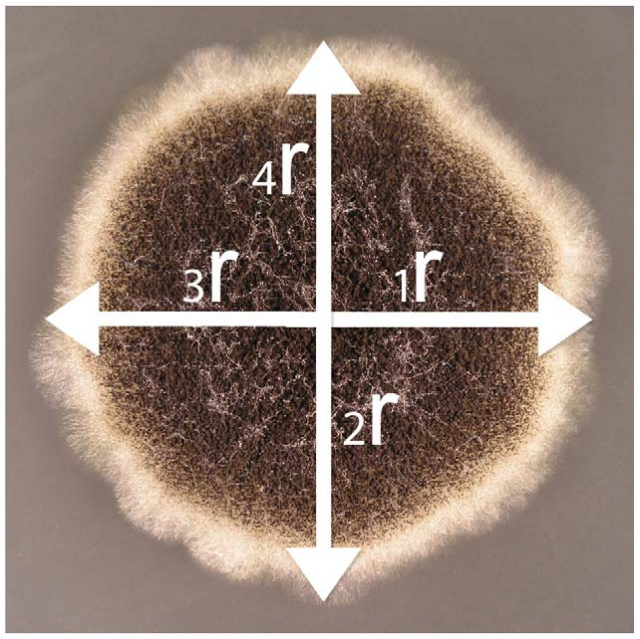
Measuring a fungal colony. The center is the sowing center, where two perpendicular lines intersect and pass to the colony edges. The four radii were measured with a digital caliper.

For each colony, we drew two perpendicular lines crossing at the sowing center (Figure 3). The angular orientation was arbitrary among replications within strains, but consistent among replications of the same colony (i.e., measurement error arose from the identification of the position of the sowing center and the colony edge, rather than the orientation of measurements). The size and individual asymmetry of a colony was estimated from the four radii (*x*_i_) (Figure 3). The mean of the four radii was the estimation of the colony size. Because all colonies were measured at the same age (i.e., 96 hours), it is also a measure of the growth rate. The individual asymmetry, which is simply the within-colony radius variation, was estimated by the Mean Absolute Difference (MAD) of *x*_i_ for each colony [5]. This is identical to Levene's test for comparing variances, and is analogous to the mean of |*R*−*L*| for bilaterally symmetrical organisms. The individual asymmetry of a single colony is the expectation of |
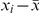
|. Thus, 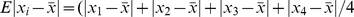. All measurements were taken from the scanned photograph using digital calipers (resolution of 0.01 mm). Each colony was measured twice. The fluctuating asymmetry of a species on a particular slope was the mean of the individual asymmetries. As a measure of effect size, we used the standardized mean difference, Hedges' *g* = 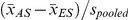, where 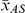 is the MAD on the “African” slope, 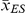 is the MAD on the “European” slope, and *s*_pooled_ is the pooled standard deviation.

### Measurement error

Measurement error (*s* ^2^_me_) inflates estimates of fluctuating asymmetry and complicates corrections for size scaling [41], [42]. Measurement error, between replicate estimates of colony asymmetry, accounted for 57.6% of the random variation (not counting the variation among slopes and species) and 41.5% of the total variation (including slopes and species). While high, this level of error is not so high as to obscure estimates of fluctuating asymmetry, which accounted for 43.4% of the random variation. The variance components for fluctuating asymmetry were greater than zero [5]. Moreover, measurement error was unavoidable because of the relative uniformity of the center of 96-h colonies, and the associated ambiguity in identifying the original sowing center.

### Correcting Asymmetry for Size Effects

If fluctuating asymmetry depends on colony size, the comparison of different species, or different populations of individual species, may be confounded with size. Positive size-scaling of asymmetry is largely due to multiplicative error associated with the active-tissue model of growth [5], [42]. For bilateral traits, this can be corrected by either dividing |*R*−*L*| by the trait mean (*R*+*L*)/2 [43], or by simply using |log *R*−log *L*| [11], [42]. The corresponding corrections for radial characters involve dividing |*x_i_*−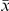
| by trait size (the mean, or expectation *E*, of *x*_i_), or by using |log *x_i_*−log
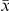
|. In practice, however, both transformations often generate negative scaling (over-correction), because measurement error is additive, not multiplicative. The mixture of multiplicative and additive errors leads to this overcorrection. An alternative recourse is the power transformation of the raw data [44], where *y*(λ) = (*y* ^λ^−1)/λ for λ≠0 and *y*(λ) = log *y* for λ = 0. A power transformation can accommodate a linear transform (λ = 1), a log transform (λ = 0), and everything in between [5], [9]. A square root transform, for example, is possible when λ = 0.5. We designate the absolute value of the transformed asymmetry value as |*d*′| = |[(*x_i_* ^λ^−1)/λ]−[(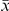 ^λ^−1)/λ]|.

To find the best value of λ for each power transform on each species that shows negative or positive size scaling, we regressed the unsigned asymmetry 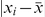 on trait size [*E*(*x_i_*)], separately for each slope and pooled across slopes. Then, we regressed | log 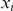
−log 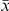
| on *E*(log *x_i_*) to look for negative size scaling associated with the traditional transformation. Finally, we regressed |[(*x_i_* ^λ^−1)/λ]−[(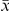 ^λ^−1)/λ]| on *E*[(*x_i_* ^λ^−1)/λ] and selected different values of λ to minimize the slope of the regression [5], [9]. We found that λ = 0.35 removes most of the relationship between size and variation in all five species, separately and together.

### Experimental design and data analysis

We used a crossed design with two levels of nesting. Slope and species were the main fixed effects. Strain within each combination of slope and species was a random effect, as were individual colonies within each strain and replicate measurements of each colony. We used a factorial ANOVA to test the main effects and their interaction. The dependent variables were the colony radii (size) and the size-corrected Mean Absolute Deviation (MAD) of the colony radii (fluctuating asymmetry).

## Results

One species of soil microfungus (*P. roseopurpureum*) was significantly more abundant on the “European” slope of “Evolution Canyon.” Moreover, *E. nidulans* was marginally more abundant on the “European” slope. The other four species were equally abundant on both slopes (Table 1).

**Table 1:** Percent relative abundance of microfungal species in the soil of the “African” (AS) and “European” (ES) slopes of “Evolution Canyon.”

Species	Abundance_AS_	Abundance_ES_	*P* (Chi-square)
*Emericella nidulans*	4.5	12.3	0.057
*Penicillium roseopurpureum*	6.8	24.5	0.0016
*Aspergillus terreus*	5.3	3.8	0.619
*Penicillium lanosum*	28.6	33.2	0.558
*Clonostachys rosea*	10.5	12.6	0.663

**Figure 4:**
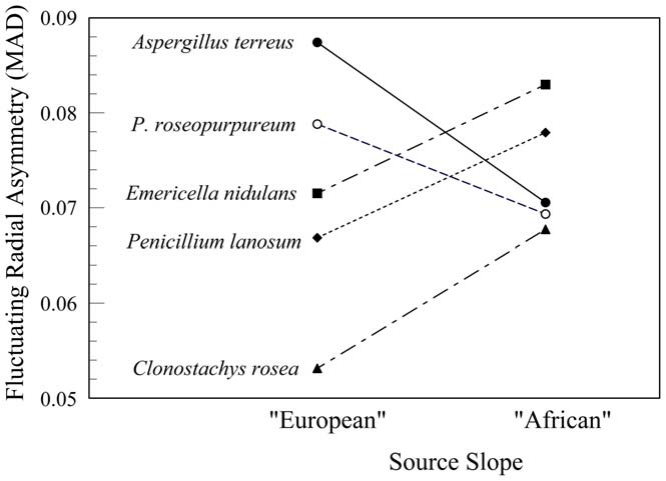
Fluctuating radial asymmetry of colonies of soil microfungi, Mean Absolute Deviation (MAD). MAD = *E*|[(*x_i_* ^λ^−1)/λ]−[(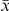 ^λ^−1)/λ]|, where *x_i_* is the radius *i* (mm) and λ = 0.35.

Fluctuating radial asymmetry differed among species (*F*_4, 48.01_ = 3.485, *P* = 0.02), but not between strains of those species from the opposing slopes of “Evolution Canyon” (*F*_1, 54.267_ = 0.416 *P* = 0.52). Colonies of *C. rosea* were more symmetrical than colonies of the other four species (Student-Newman-Keuls, *P*<0.05) (Figure 4). Nevertheless, there was a marginally insignificant slope by species interaction (*F*_4, 46.869_ = 2.500, *P* = 0.055). Colonies of *E. nidulans*, *P. lanosum*, and *C. rosea* from the “African” slope were more asymmetric than those from the “European” slope, while colonies of *A. terreus* and *P. roseopurpureum* from the “European” slope were more asymmetric than those from the “African” slope (Figure 4). Only *C. rosea* showed significant differences between “African” and “European” slopes (the “African” strains were more asymmetric, *F*_1, 12.247_ = 5.114, *P*<0.05). None of the other four species showed significant differences between strains from the two slopes (*F*_1, 5.864-14.884_ = 0.163–2.741, *P*≥0.135).

**Figure 5:**
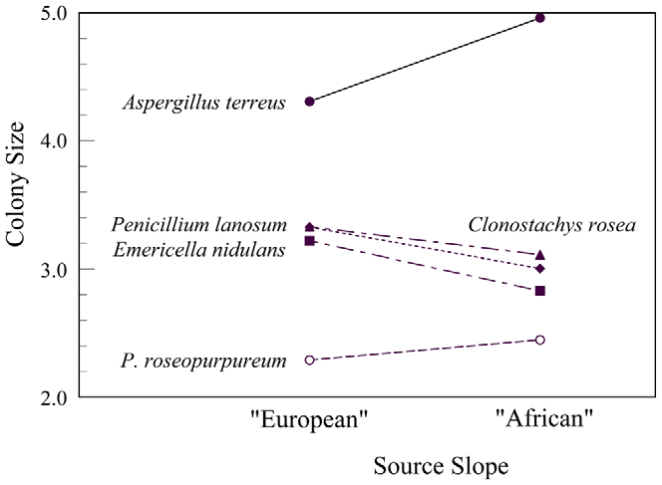
Mean size of colonies of soil microfungi. *E*[(*x_i_* ^λ^−1)/λ], where *x_i_* is the radius *i* (mm) and λ = 0.35.

Mean colony size differed among species (*F*_4, 51.873_ = 102.122, *P*<0.001), but not between strains of those species from the opposing slopes of “Evolution Canyon” (*F*_1, 53.249_ = 0.410, *P*>0.520). The sizes of the colonies, ranked from smallest to largest are *P. roseopurpureum*<*E. nidulans*<*P. lanosum* = *C. rosea*<*A. terreus* (Student-Newman-Keuls, *P*<0.05) (Figure 5). The interaction between slope and species, however, was highly significant (*F*_4, 51.613_ = 5.692, *P*<0.002). Colonies of *A. terreus and P. roseopurpureum* from the “African” slope were larger than those from the “European” slope, while colonies of *C. rosea*, *P. lanosum*, and *E. nidulans* from the “European” slope were larger than those from the “African” slope (Figure 5). Only *P. lanosum* showed significant differences between “African” and “European” slopes (*F*_1, 14.197_ = 10.841, *P*<0.006). Strains from the “European” slope were larger than those from the “African” slope. Strains of *E. nidulans*, *P. roseopurpureum*, and *C. rosea* from the opposing slopes showed marginally insignificant differences in size (*F*_1, 8.113-13.011_ = 3.645–4.631, *P* = 0.051–0.075). Strains of *E. nidul*ans and *C*. *rosea* from the “European” slope were larger than those from the “African” slope, while strains of *P. roseopurpureum* from the “African” slope were larger than those from the “European” slope. Strains of *A. terreus* showed no differences between the two slopes (*F*_1, 6.972_ = 2.488, *P*>0.155).

**Figure 6:**
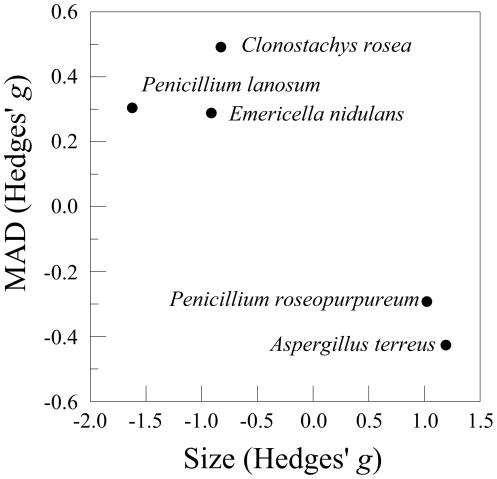
Scatter plot of fluctuating asymmetry (MAD) versus the difference in colony size between the “European” and “African” slopes (Hedges' *g*).

There was a significant negative correlation between the effect sizes for fluctuating radial asymmetry and mean colony size (*r* = 0.923, *df* = 3, *P*<0.05, Figure 6). Differences in colony asymmetry between the two slopes were inversely related to differences in growth rate; strains from the slope exhibiting reduced growth exhibited greater asymmetry. However, the strength of this relationship is not known with certainty, due to the small number of species examined.

## Discussion

This is the first study to examine fluctuating asymmetry of microorganisms, namely soil microfungi, as an ecological indicator. We grew soil microfungi under controlled conditions in the laboratory. The common-garden experiments allow us to quantify the heritable components of phenotypic variation [45]. Thus, interaction of slope and species in both growth rates and asymmetry of microfungi in a common environment is evidence of genetic differences between the microfungal strains from the “African” and “European” slopes of “Evolution Canyon.” Moreover, interslope differences in fluctuating radial asymmetry of *C. rosea* strains suggest that the strains from the “African” slope are less well adapted to the conditions of the laboratory environment.

Our previous studies showed that soil microfungi were reliable indicators of environmental stress at “Evolution Canyon” [46], [47]. The “African” slope community was characterized by higher diversity (species richness, Shannon index, and evenness) [47]. Moreover, there was a strong interslope impact of edaphic (soil-related) and microclimatic conditions (mainly moisture levels) on the pattern of microfungal community composition.

In the previous studies at “Evolution Canyon,” the wild barley, *Hordeum spontaneum*, showed adaptation to the opposing slopes [48]. All populations developed faster in the sun, but this trend was more evident in the plants from the “African” slope, whose development in the shade was slower than those originating from the “European” slope. In addition, the mutation rate under mild laboratory conditions and the survival ability of *Aspergillus niger* were higher on the “African” slope [49], [50]. All these findings suggest that differences in asymmetry and size, which we observed between strains of the same microfungal species grown under identical laboratory conditions, reflect differences between the opposing slopes of “Evolution” Canyon in the wild.

Our study suggests that fluctuating radial asymmetry has potential as an indicator of random phenotypic variation and stress in soil microfungi. Nevertheless, most of the species-specific differences in asymmetry between the slopes were insignificant (except for *C. rosea*). However, with more species, the small differences between experimental groups might attain statistical significance more broadly. The statistically significant interaction between slope and species suggests that, as a group, the strains from “European” and “African” slopes respond differently to the lab environment. Within a single species, fluctuating asymmetry is generally sensitive only to severe stress [7], but by aggregating data for several species, the effects of less severe stress can nevertheless be detected. In future studies, we intend to quantify fluctuating asymmetry and growth of soil microfungi from “African” and “European” slopes under a range of laboratory conditions, with varying temperature, radiation, and humidity. Likewise, we plan to increase the number of species, and compare patterns between the canyon at Nahal Oren and other canyons.
